# Chitosan Oligosaccharides Regulate the Occurrence and Development of Enteritis in a Human Gut-On-a-Chip

**DOI:** 10.3389/fcell.2022.877892

**Published:** 2022-04-26

**Authors:** Bolin Jing, Kun Xia, Chen Zhang, Siming Jiao, Limeng Zhu, Jinhua Wei, Zhuo A. Wang, Nannan Chen, Pengfei Tu, Jianjun Li, Yuguang Du

**Affiliations:** ^1^ State Key Laboratory of Biochemical Engineering, Institute of Process Engineering, Chinese Academy of Sciences, Beijing, China; ^2^ State Key Laboratory of Natural and Biomimetic Drugs, School of Pharmaceutical Sciences, Peking University, Beijing, China; ^3^ The Second Affiliated Hospital, Hengyang Medical School, University of South China, Hengyang, China; ^4^ China Food Industry Promotion Center, Beijing, China

**Keywords:** chitosan oligosaccharides, human gut-on-a-chip, enteritis, mucous layer, *E. coli*

## Abstract

Past studies on the protective effects of chitosan oligosaccharides (COS) on inflammatory bowel disease (IBD) commonly rely on animal models, because traditional cell culture systems couldn’t faithfully mimic human intestinal physiology. Here a novel human gut-on-a-chip microsystem was established to further explore the regulatory effects of COS on the occurrence and development of human enteritis. By constructing an intestinal injury model caused by dextran sodium sulfate (DSS) on the chip, this study proved that COS can reduce intestinal epithelial injury by promoting the expression of the mucous layer for the first time. By establishing an inflammatory bowel disease model on the chip caused by *E. coli* 11775, this study demonstrated that COS can protect the intestinal epithelial barrier and vascular endothelial barrier by inhibiting the adhesion and invasion of *E. coli* 11775 for the first time. In addition, similar to the results *in vivo,* COS can decrease the inflammatory response by reducing the expression of toll-like receptor 4 protein and reducing the nuclear DNA binding rate of nuclear factor kappa-B protein on this chip. In summary, COS can be used as a potential drug to treat human IBD and the human gut-on-a-chip would be used as a platform for quick screening drugs to treat human IBD in future.

## Introduction

Enteritis is a disease with a high incidence in both developed and developing countries. As the most common type of enteritis, inflammatory bowel disease (IBD) has become a global problem and mainly includes two types: ulcerative colitis and Crohn’s disease (Kaser et al., 2010). Ulcerative colitis is a chronic nonspecific inflammatory disease that invades the colonic mucosa and is a long-term inflammatory response. Crohn’s disease is a granulomatous inflammatory disease that combines fibrosis and ulcers and can affect the entire gastrointestinal tract anywhere (Fiocchi, 1998). Intestinal barriers and inflammation have been proven in past studies to be the main regulators of the occurrence and development of IBD (Garrett et al., 2010; Shin and Kim, 2018). Intestinal barriers include microecological, mucus barriers and intestinal epithelial barriers. Immunomodulation includes specific antigen recognition, immune responses and cell autophagy (Artis, 2008). Regulating intestinal microbiome homeostasis, reducing intestinal damage and inhibiting inflammation are the main strategies for treating enteritis (Hooper and Macpherson, 2010; Maloy and Powrie, 2011).

Chitosan oligosaccharides (COS) are s formed by glucosamines linked through β-1,4 glycosidic bonds with a degree of polymerization (DP) of 2–10. COS have anti-inflammatory functions, and promote the proliferation of beneficial bacteria such as bifidobacteria and lactic acid bacteria, and can be used in medicine, functional foods and other fields (Liaqat and Eltem, 2018). Yousef et al. (2012) demonstrated that COS can effectively reduce the damage and inflammation of enteritis caused by dextran sodium sulfate (DSS) at a concentration of 10–20 mg/kg/day. Bahar et al. 2012 demonstrated that COS can regulates the expression of IL-8 in Caco2 cells through the NF-κB signaling pathway and the AP-1 signaling pathway. Our group proved that COS can reverse the dysbiosis of gut microbiota and protect the barrier function in diabetic mice in a past study (Zheng et al., 2018a). However, most of these studies are based on animal models or simple cell culture systems, and there are fundamental differences between animals and humans. Therefore, it is difficult to reproduce the enteritis microenvironment by using simple cell culture systems (David et al., 2014; Ma et al., 2021). The regulation of COS on the occurrence and development of human enteritis has still rarely reported on a human IBD model.

The human gut on a chip is a microfluidic cell culture system that recapitulates the structure, function, physiology, and pathology of living human intestine *in vitro*. Past studies have demonstrated that by using the human gut on a chip, the drug activity and mechanism of enteritis can be studied (Shah et al., 2016; Bein et al., 2018; Nelson et al., 2021). Kim et al. (2016) introduced conditional pathogenic *E. coli* and human monocyte macrophages into the human intestinal chip to construct a corresponding pathological model of enteritis and observed gut inflammation, such as mucous layer damage, barrier damage, and high expression of inflammatory factors. Later, Shin and his coworkers introduced dextran sodium sulfate (DSS) in the intestinal cavity to construct a human enteritis model for the first time *in vitro* (Shin and Kim, 2018). In a recent study, we also constructed a peristaltic intestine on a chip, and we also proved that *Lactobacillus casei* L5 can inhibit the occurrence and development of enteritis induced by *E. coli* in our human gut microsystem (Jing et al., 2020).

In this study, we hypothesize that COS have beneficial effects on the occurrence and development of enteritis in a human gut model. Here, we constructed a human gut IBD model on a chip, which includes microbes, mucous layer, intestinal epithelial barrier, vascular endothelial barrier and immune cells, to further explore the regulatory effects of COS on the intestinal mucus layer, intestinal barrier, conditional pathogen and inflammatory response during human enteritis.

## Materials and Methods

### Preparation of COS

COS were prepared by enzymatic hydrolysis of chitosan and separated with a membrane as described in a previous study (Zheng et al., 2018b).

### Cells Culture

Human intestinal epithelial Caco2 cells from ATCC (New York, United States) were cultured in Dulbecco’s modified Eagle’s medium containing 4.5 g*L^−1^ glucose medium (DMEM/HG) supplemented with 10% (w/v) fetal bovine serum (FBS). Primary human umbilical vein endothelial cells (HUVECs) (Lonza, Basel, Switzerland) were cultured in ECM medium (DMEM/F12) containing 5% (w/v) FBS. Primary human macrophage cells (OriCells, Shenyang, China) were cultured in RPMI 1640 medium containing 10% (w/v) FBS. Penicillin (100 units/ml) and streptomycin (100 μg/ml) were added to all aforementioned media. All cells were cultured in a cell incubator with 5% CO_2_ at 37°C. Antibiotics were removed from the culture media for coculture of human cells with living intestinal microbes. All cell culture media were purchased from Thermo Fisher Scientific (Waltham, MA, United States). Caco2 cells were used in generations 2–10, and HUVECs were used in generations 2–6 and macrophages were used in the first passage.

### Fabrication and Assembly of the Microfluidic Chip

The peristaltic three-dimensional human gut microsystem used in this study was fabricated from polydimethylsiloxane (Dow Corning, Michigan, United States), described as previously (Jing et al., 2019a; Jing et al., 2019b). The polydimethylsiloxane (PDMS) plates, which were developed by Whitesides et al., were fabricated by soft lithography using photoresist as the template. Briefly, all microchannel layers were individually prepared by casting PDMS prepolymer (10:1 w/w ratio of PDMS to curing agent) on a microfabricated mold of the inverse channel design made of photoresist and curing the polymer at 60°C for 12 h. After the microchannel layers were peeled from the wafer, peripheral holes (1.5-mm diameter) for tubing were punctured on the PDMS plates. Additionally, 5-mm-diameter holes were made in the center of each PDMS plate to facilitate connections between the endothelial monolayer and the intestinal epithelial cells. Porous PDMS membranes with a 5-mm diameter and 10-μm pore size were placed between PDMS plates for on-chip cell culture (Supplementary Figure S1A). After careful alignment along the vertical direction, the PDMS plates were superimposed with the top and bottom polymethyl methacrylate (PMMA) frames and fastened with screws (Supplementary Figure S1B). Polyvinyl chloride (Watson-Marlow, Buckinghamshire, England) tubes with an inner diameter of 25 μm were connected from two medium tanks with a multichannel pneumatic pump (FUIGENT, France) to the upper medium and lower microfluidic channels (Supplementary Figure S1C). This allowed us to regulate the application of air pressure to the fluid medium by a computer to exert cyclic changes of fluid pressure within each of the microchannels to mimic peristaltic motions.

### Establishing the Inflammatory Bowel Disease Model Caused by *E. coli* 11775 on the Chip

This model was constructed in a similar way as we have reported (Jing et al., 2020). Before the microdevice was assembled, the porous membrane was coated with collagen type I hydrogel at 37°C for 1 h. The Caco-2 cells (1 × 10^5^ cells/cm^2^) were seeded on the porous PDMS membrane and incubated at 37°C for 24 h, allowing the seeded intestinal epithelial cells to attach to the membrane surface. Then, HUVECs (1 × 10^5^ cells/cm^2^) were seeded onto the basal of the membrane and incubated at 37°C for 4 h. After vascular endothelial cells attached to the membrane, the microdevice was assembled and culture medium was perfused into microchannels at 60 μl/h with cyclic peristalsis (15%, 0.17 Hz) for 5 days. *Escherichia coli* 11775 strain was purchased from the China General Microbiological Culture Collection Center (CGMCC, Beijing, China). It was cultivated in autoclaved LB medium (Gibco, MA, United States) at 37°C and 200 rpm for 12 h. After the bacterial cell density was adjusted to ∼1.0 × 10^6^ CFU/ml, *E. coli* was spun down (10,000 g, 8 min), and resuspended in antibiotic-free DMEM with 5 μmol/ml red cell tracker (Invitrogen, MA, United States), and cultivated at 37°C for 30 min. Then, *E. coli* was spun down, and resuspended in antibiotic-free DMEM plus 10% FBS medium immediately, and flowed into the intermediate microchannel containing the villus epithelium precultivated in the microfluidic chip with peristalsis plus vascular endothelial cells for 4 days. At the same time, macrophages (5 × 10^4^ cells/cm^2^) were introduced into the vascular lumen. After *E. coli* cells and macrophages cells were allowed to attach to the surface of the epithelium or endothelium for ∼2 h under static conditions, fresh antibiotic-free medium was perfused into microchannels at 60 μl/h with cyclic peristalsis (15%, 0.17 Hz).

### Morphological Studies

Morphological observation was performed by following the standard protocol. After cultivation, the cells were fixed with 4% paraformaldehyde for 15 min, and permeabilized with 0.1% Triton X-100 (Thermo Fisher, MA, United States) in PBS for 10 min, and blocked with 3% BSA (Thermo Fisher, MA, United States) in PBS for 30 min at room temperature. To visualize epithelial tight junctions, cells were incubated with occludin monoclonal antibody (Thermo Fisher Scientific, MA, United States) at 10 μg/ml in blocking buffer for 1 h at room temperature and washed with PBS. To visualize the proteins of intestinal microvilli, occludin or NF-κB (p65), cells were incubated with villin polyclonal antibody (Invitrogen, MA, United States) at 10 μg/ml in blocking buffer, occludin monoclonal antibody (331588, Invitrogen) at 5 μg/ml or NF-κB (p65) monoclonal antibody (Abcam, Cambridge, United Kingdom) for 1 h at room temperature, and washed with PBS, incubated with goat anti-rabbit IgG (H + L) superclonal secondary antibody and Alexa Fluor^®^ 594 conjugate (Invitrogen, MA, United States) at a dilution of 1:1,000 for 2 h at room temperature. To visualize the carbohydrates of the intestinal epithelial mucus layer, cells were incubated with fluorescein isothiocyanate-labeled wheat germ agglutinin (Sigma, MO, United States) for 30–45 min at 37°C and washed with PBS. To visualize the VE-cadherin protein in HUVECs, cells were incubated with cd144 polyclonal antibody (Invitrogen) at 5 μg/ml in blocking buffer at 5 μg/ml for 1 h at room temperature. The nuclei were stained blue with Dapi. Images were taken under an inverted laser scanning confocal microscope (Zeiss, Oberkochen, Germany).

### Measurement of Paracellular Permeability

The barrier capacity of the intestinal-vascular coculture layers was evaluated by measuring the apparent permeability (*P*_*app*_) of FITC-labeled dextrans with molecular weight of 4 and 40 kDa (Sigma, MO, United States) through the coculture layer. One milliliter of Hank’s Buffered Salt Solution (HBSS) containing FITC-dextrans (2 nmol/ml) was perfused through the microchannel of the top layer, and the blank HBSS was circulated through the lower microchannel at a flow rate of 60 μl/h. *P*_*app*_ was calculated using the equation below:

$$papp\left(cm/s\right)=\left(Coutput\left(mg/ml\right)\times flowrate\left(ml/s\right)\right)/\left(Cinput\left(mg/ml\right)\times A\left(cm^{\wedge }2\right)\right)$$

where *A* = area of mass transfer, *Cinput* = donor concentration of reagent in the upper microchannel, *Coutput* = concentration of reagent in the lower microchannel and *flowrate* = 1.7 × 10^−5^ *ml/s.*

### Inflammatory Responses

To analyse inflammatory cytokines, medium was collected from the outlet of both the intestinal cavity and vascular lumen and then immediately frozen at −80°C until the analysis was performed. The amounts of cytokines (IL-8, IL-6, TNF-α and IL-1β) in each sample (*n* = 3) were analysed using ELISA kits (Thermo Fisher Scientific, MA, United States) according to the manufacturer’s protocol.

### Data Analysis and Quantification

All data were analysed by averaging the values of at least three microfluidic devices, with each device representing one independent experiment. All results and error bars in this article were represented as the mean ± SD. One-way ANOVA or Student’s tests (*n* = 3, *p* < 0.05) were performed with GraphPad Software.

## Results

### Characteristics of the Human Gut-on-a-Chip

**FIGURE 1:**
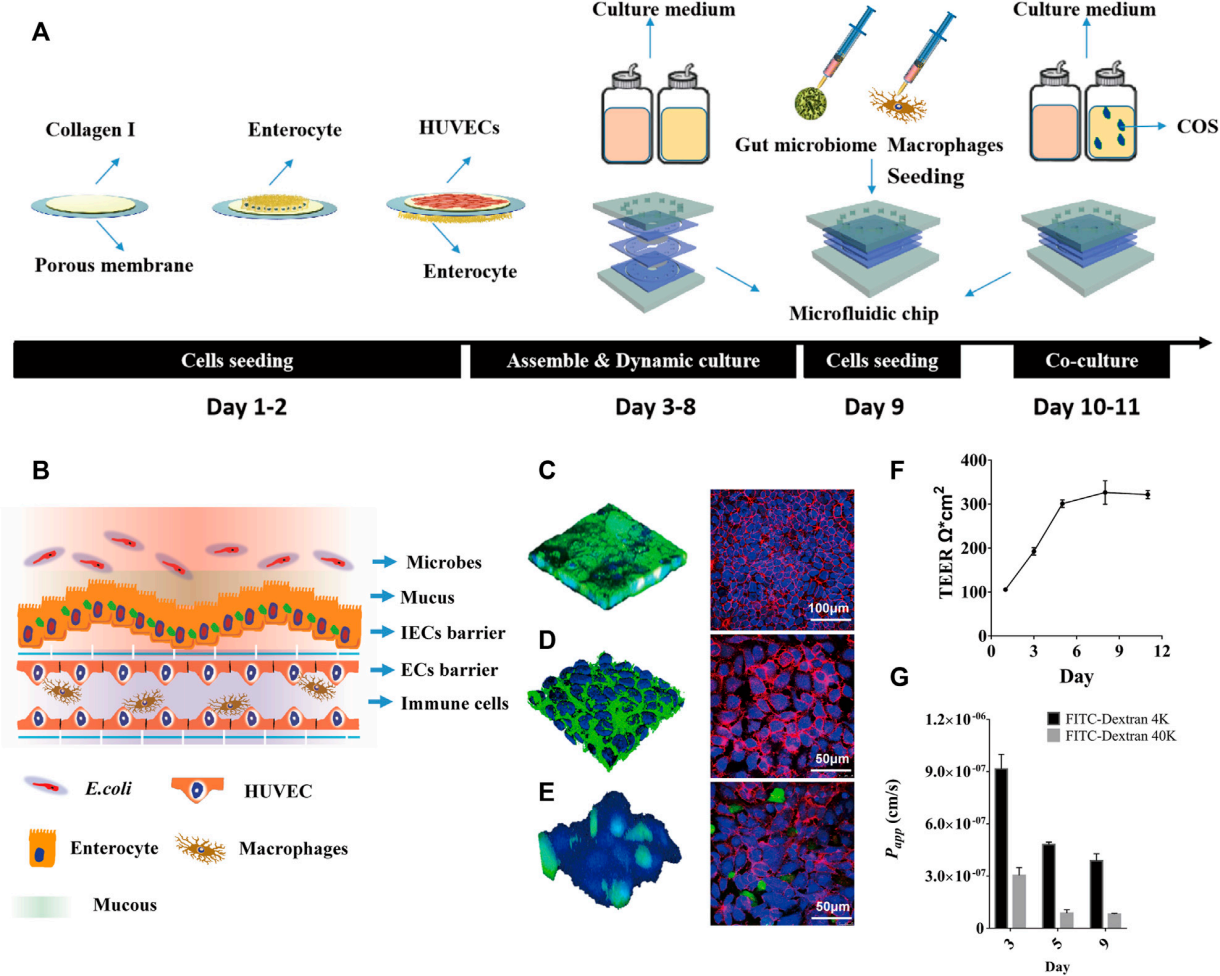
The human inflammatory bowel disease model on a chip. **(A)** Diagrammatic overview of the construction protocol of inflammatory bowel disease model on a chip. **(B)** Schematic diagram of human gut-on-a-chip with microbes, mucus, intestinal epithelial cells (IECs) barrier, endothelial cells (ECs) barrier and immune cells. **(C)** Intestinal mucin staining using wheat germ agglutinin (WGA) labeled with FITC (Left, Green), and immunofluorescence staining against occludin on intestinal epithelial cells (Right, Red); **(D)** Vascular endothelial glycocaly staining using WGA-FITC (Left, Green), and immunofluorescence staining against CD144 (VE-cadherin) on endothelial cells (Right, Red). **(E)** Macrophages (Green) adhering to the vascular endothelial cells (Blue) surface, and immunofluorescence staining against CD31 (PECAM-1) on endothelial cells (Right, Red). **(F)** TEER values of IECs co-cultured with HUVECs on the chip for 11 days. **(G)** The *P*_*app*_ value of FITC-labeled dextran (4 kDa, 40 kDa) through IECs co-cultured with HUVECs on the chip.

Here, a laminated microfluidic chip was used to construct the human inflammatory bowel disease model, which was similar to ones used in our past studies (Jing et al., 2019a; Jing et al., 2020), including two layers of PDMS chips, two layers of elastic porous membranes and two layers of PMMA plates (Supplementary Figure S1A,B). The porous membrane with a thickness of 30 μm and a pore diameter of 10 μm was coated with Collagen Type I. By sequentially inoculating intestinal epithelial cells, vascular endothelial cells, and macrophages on the porous membrane, a coculture system of human gut inflammation on a chip was constructed (Figure 1A). After 5 days of culture under fluid dynamic conditions, the intestinal lumen can differentiate into a mucous layer and a dense intestinal epithelial barrier, the vascular lumen can form a dense vascular endothelial barrier and an immune microenviroment (Figure 1B–E).

By labeling intestinal epithelial cells with FITC-WGA and FITC-occludin, it was seen that the intestinal cavity forms an undulating mucus layer and dense intercellular connections (Figure 1B). By labeling vascular endothelial cells with FITC-WGA and FITC-CD144 (VE-cadherin), the glycocalyx secreted by the vascular endothelial layer and dense intercellular junctions were observed (Figure 1C). By labeling macrophages with green celltracker and vascular endothelial cells with blue celltracker and CD31 (PECAM-1), it was observed that a portion of macrophages adhered to the surface of vascular endothelial cells (Figure 1D). After 5 days of dynamic culture, the resistance value of the co-culture model could reach 305 Ω*cm^2^ and remained stable for a week (Figure 1E). During dynamic cultivation, the apparent permeability of FITC-40 kDa dextran through the intestinal epithelial layer was significantly lower than that of FITC-4 kDa dextran (Figure 1F). After 5 days of dynamic culture, the *Papp* values of FITC-4 kDa dextran and FITC-40 kDa dextran could reach 4.5*10^−7^ cm/s and 8.1*10^−8^ cm/s, and remained stable for a week.

### COS Regulate the Mucus Layer in Human Gut Injury Model Induced by DSS

**FIGURE 2:**
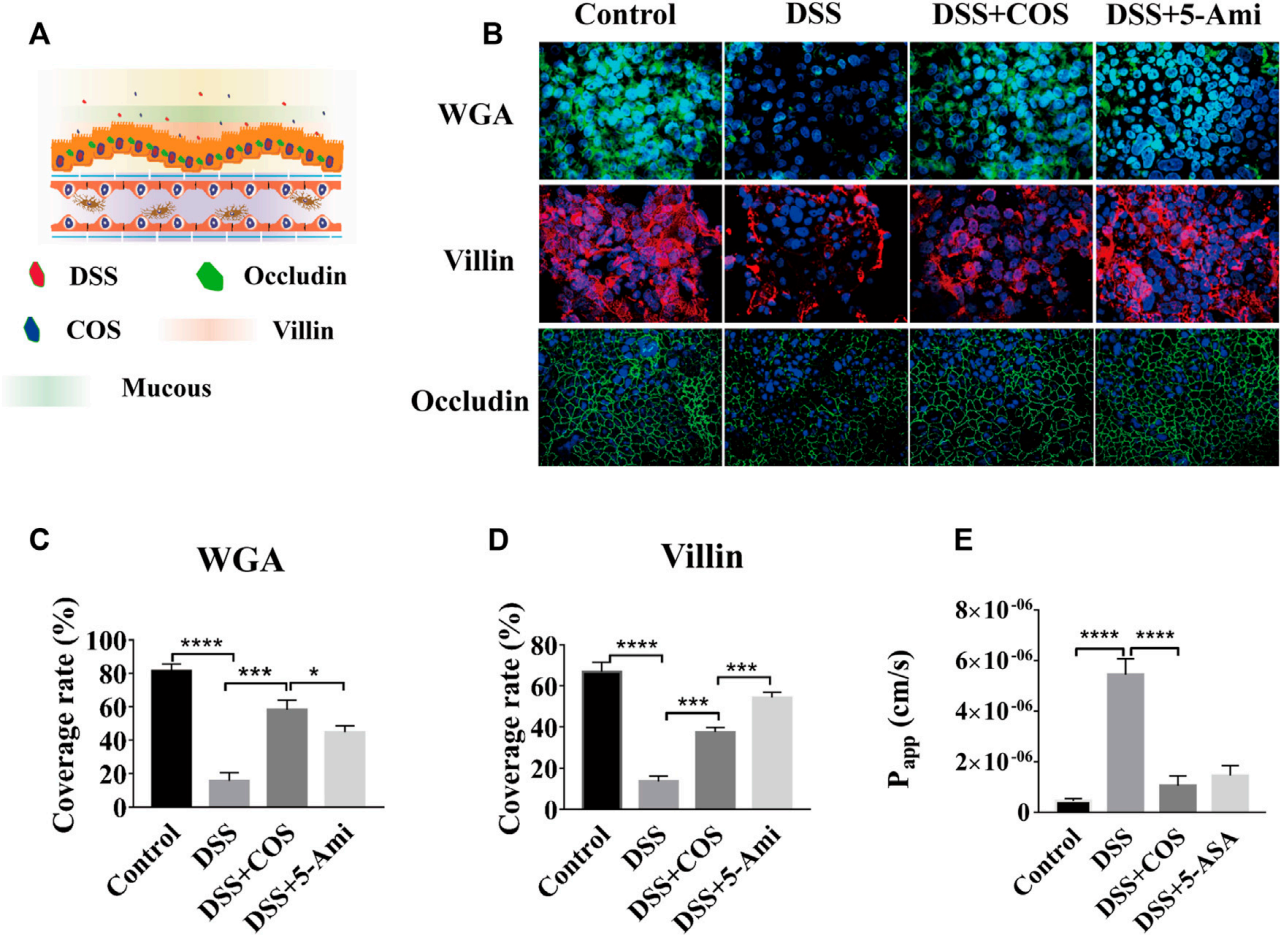
Effects of COS on injury of gut barrier induced by DSS. **(A)** Schematic diagram of a model of human gut injury induced by DSS. **(B)** Intestinal mucin staining using WGA-FITC (Green), and immunofluorescence staining against villin (Red) and occludin (Green) on intestinal epithelial cells (Green). **(C)** The coverage of mucin on IECs. **(D)** The coverage of villin on IECs. **(E)** The apparent permeability of FITC-dextran (4 kDa) across intestinal epithelium. Data were represented as mean ± SD (*n* = 3); One-way ANOVA test was performed, *p < 0.05, ***p < 0.001, ****p < 0.0001.

To investigate the repair effect of COS on damage of intestinal epithelial mucosal and barrier function, DSS treatment was performed in the intestinal cavity for 24 h to build a DSS inflammation injury model. Then COS and 5-aminosalicylic acid (5-ASA) were added respectively and incubated for another 24 h (Figure 2A). 5-ASA was a commonly used positive drug in the treatment of ulcerative colitis. Through morphological analysis, it was seen that DSS can cause damage to the mucus layer and villin of intestinal epithelial cells. After being treated with COS and 5-ASA, both the mucus layer and villin were effectively repaired and tended to return to normal (Figure 2B). Statistical analysis showed that the repair effect of COS on coverage of mucous layer was slightly better than 5-ASA, 58.3% (COS) versus 54.4% (5-ASA). (Figure.2C–D). Then, the effects of COS on the expression of the mucous layer at different times were monitored to further explore whether COS can promote the expression of the mucous layer in the intestinal epithelium. Morphological characterization and statistical analysis showed that COS had a significant promotion effect on the expression of the intestinal epithelial mucous layer (Supplementary Figure S2A). Compared with the control group, the coverage of intestinal epithelial mucosa increased by approximately 30% after treatment with COS for 48 h, while the adhesion rate of COS marked by FITC was only approximately 2% (Supplementary Figure S2B). By labeling intestinal epithelial cells intercellular connexin with FITC-occludin and measuring apparent permeability of FITC-dextran (4 kDa) across intestinal epithelium, it was found that COS had significant repair effects on damage of intestinal epithelial barrier (Figure 2B). After treatment of intestinal epithelial cells with DSS for 24 h, the apparent permeability of FITC-4 kDa dextran through the intestinal epithelial layer was 5.4*10^−6^ cm/s. After treatment with COS or 5-ASA for another 24 h. The permeability returned to the normal level, reaching 8.5*10^−7^ cm/s and 1.4*10^−6^ cm/s, respectively (Figure 2E).

### COS Regulate the Microbial Homeostasis in Enteritis Model Induced by *E. coli* 11775

**FIGURE 3:**
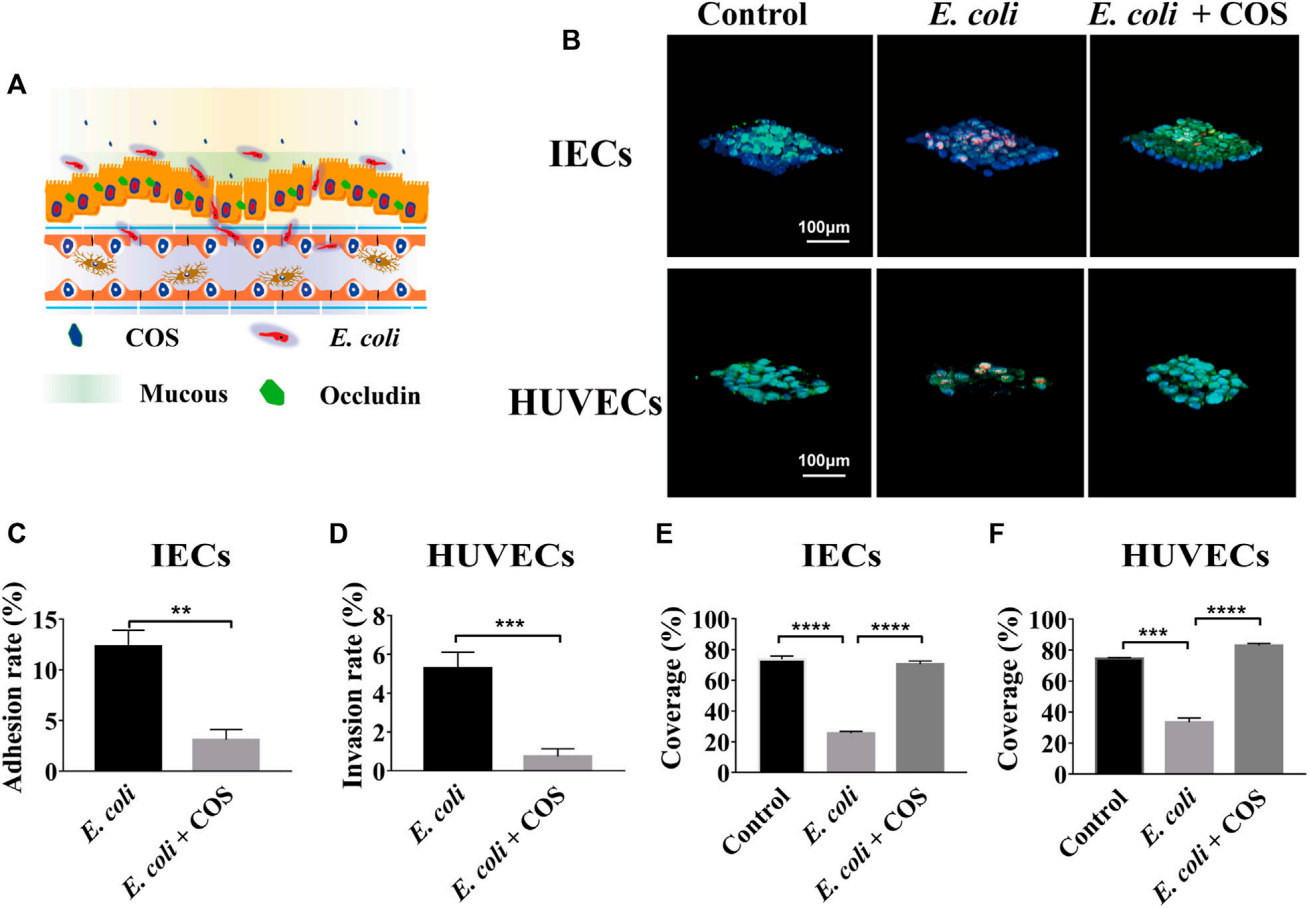
Effects of COS on human enteritis induced by *E. coli* 11775. **(A)** Schematic diagram of human enteritis model induced by *E. coli* 11775. **(B)** Intestinal mucin and vascular endothelial glycocaly staining using WGA-FITC (Green), intestinal epithelial cells (IECs) an endothelial cells (ECs) staining using blue celltracker, and *E. coli* 11775 staining using red celltracker. **(C)** The adhesion rate of *E. coli* 11775 to IECs. **(D)** The invasion rate of *E. coli* 11775 to ECs. **(E)** The coverage of mucus on IECs. **(F)** The coverage of glycocaly on ECs. Data were represented as mean ± SD (*n* = 3); One-way ANOVA test and Student’s test were performed, *p < 0.05, ***p < 0.001, ****p < 0.0001.

To investigate the regulatory effect of COS on human intestinal inflammation caused by intestinal pathogenic bacteria, conditionally pathogenic *E. coli* 11775 was introduced into the intestinal cavity and cocultivated with intestinal epithelial cells for 24 h, and then COS were added and cocultured for 24 he (Figure 3A). *E. coli* 11775 was labeled with red celltracker, and Caco-2 cells and HUVECs were labeled with blue celltracker. Through morphological observation and statistical analysis, it was found that COS can effectively reduce the amount of *E. coli* 11775 adhering to intestinal epithelial cells and invading into the vascular cavity (Figure 3B). In the control group, the coverage of *E. coli* 11775 adhering to epithelial cells reached 12.3%, while the adhesion coverage of *E. coli* 11775 in the presence of COS was only 3.1% (Figure 3C). In addition, the rate of *E. coli* 11775 invasion into blood vessels reached 5.3% in the blank group, while the invasion rate of *E. coli* 11775 in the presence of COS was only 0.7% (Figure 3D). FITC-WGA was used to label intestinal epithelial mucosal and vascular endothelial glycocalyx. Through morphological and statistical analysis, it was found that COS can effectively reduce injury of intestinal epithelial mucus layer and vascular endothelial glycocalyx layer (Figure 3B). The invasion of *E. coli* 11775 reduced the coverage of intestinal epithelial mucus and vascular endothelial glycocalyx by 41.1% and 48.1%, respectively, and after treatment with COS, the coverage of the mucus layer and glycocalyx layer were restored by 50% and 45%, respectively (Figure 3E–F). FITC-occludin and FITC-CD144 were used to label the intercellular connexin of intestinal epithelial cells and vascular endothelial cells, respectively (Supplementary Figure S3A). FITC-4 kDa dextran was used to test the apparent permeability. It was observed that COS showed significant protective effects on intercellular junction proteins in the intestinal epithelium and vascular endothelium. After invasion of *E. coli* 11,775, the apparent permeability of FITC-4 kDa dextran increases to 5.9*10^−6^ cm/s, while the permeability can be maintained at 1.2*10^−6^ cm/s in the presence of COS, which is equivalent to the normal state (Supplementary Figure S3B).

**FIGURE 4:**
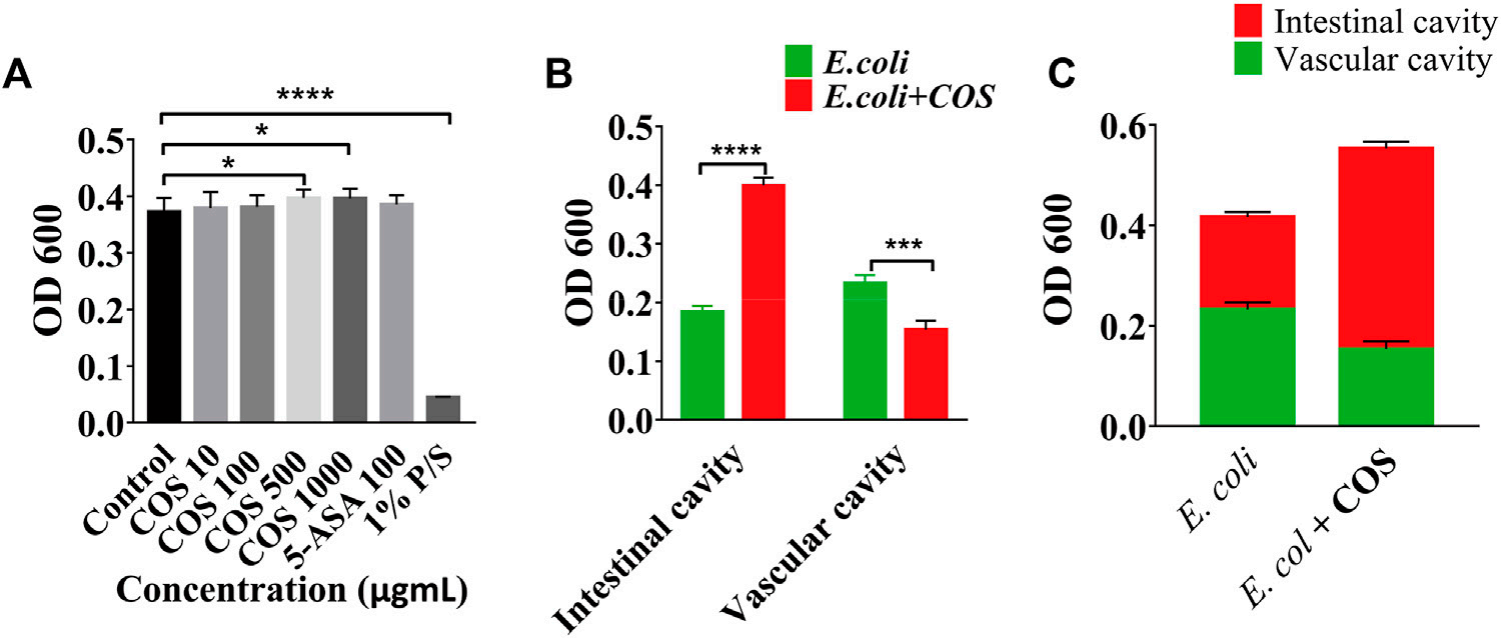
Regulatory effect of COS on microbial homeostasis in enteritis model. **(A)** Absorbance value of *E. coli* solution after treated with COS, 5-Ani or 1% P/S. **(B)** Absorbance value of outflow solution in the intestinal cavity or vascular cavity. **(C)** Absorbance value of outflow solution in the enteritis on a chip with or without COS. Data were represented as mean ± SD (*n* = 3); One-way ANOVA test and Student’s test were performed, *p < 0.05, ****p < 0.0001.

To further prove that COS can reduce adhesion and invasion of *E. coli* 11775, the amounts of *E. coli* 11775 under different culture conditions were tested. First, *E. coli* 11775 was cultured in LB medium, DMEM medium with or without COS for 24 h, there was no significant difference in the absorption value of *E. coli* solution at 600 nm (Supplementary Figure S4). After that, *E. coli* 11775 was cultured in media containing different concentrations of COS for 24 h, the absorption values of *E. coli* solutions at 600 nm were similar (Figure 4A). These indicated that neither culture media nor COS affected the proliferation of *E. coli* 11775. By testing the absorbance value of outflow solution in the intestinal cavity or vascular cavity, it was found that in the presence of COS, the concentration of *E. coli* 11775 in the intestinal efflux was higher than that without COS, and the concentration of *E. coli* 11775 in the vascular efflux was lower than that without COS (Figure 4B). In addition, the total concentration (both intestinal lumen and vascular lumen) of *E. coli* in the effluent media with COS was also higher than that without COS (Figure 4C). These results indicated that COS can reduce the amounts of adhesion and invasion of *E. coli* 11775.

### COS Reduce Inflammation Caused by *E. coli* 11775

**FIGURE 5:**
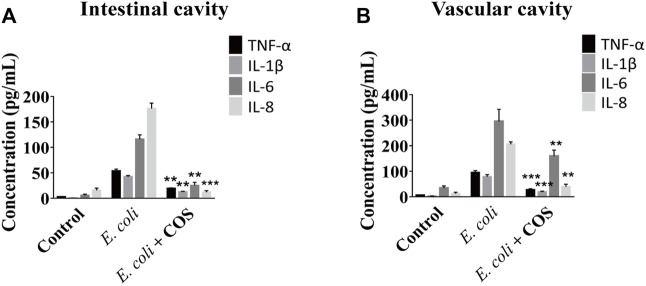
COS reduced inflammation caused by *E. coli* 11775. The concentrations of inflammatory factors in the outflow solution from intestinal cavity **(A)** or vascular cavity **(B)** on the chip. Data were represented as mean ± SD (*n* = 3); *E. coli* vs. *E. coli +* COS group, One-way ANOVA test was performed, **p < 0.01, ***p < 0.001.

In addition, the regulatory effect of COS on the inflammatory response was further explored. First, the expression levels of NO in the intestinal cavity and vascular cavities examined and it was found that the amount of NO was significantly reduced in the presence of COS, indicating that COS had a significant regulatory effect on oxidative stress (Supplementary Figure S5). Then, it was observed that COS can significantly reduce the expression levels of the inflammatory factors TNF-α, IL-1β, IL-6 and IL-8 in the intestinal cavity and vascular lumen, indicating that COS with a polymerization degree of 2–6 can significantly reduce the inflammatory response. Compared with the control group, in the presence of COS, expression level of IL-8 was decreased by 16 times in the intestinal cavity, and the expression levels of TNF-α and IL-1β were decreased by nearly 4 times in the vascular cavity (Figure 5).

### COS Regulate the NF-κB (p65) Signaling Pathway in Enteritis on a Chip

**FIGURE 6:**
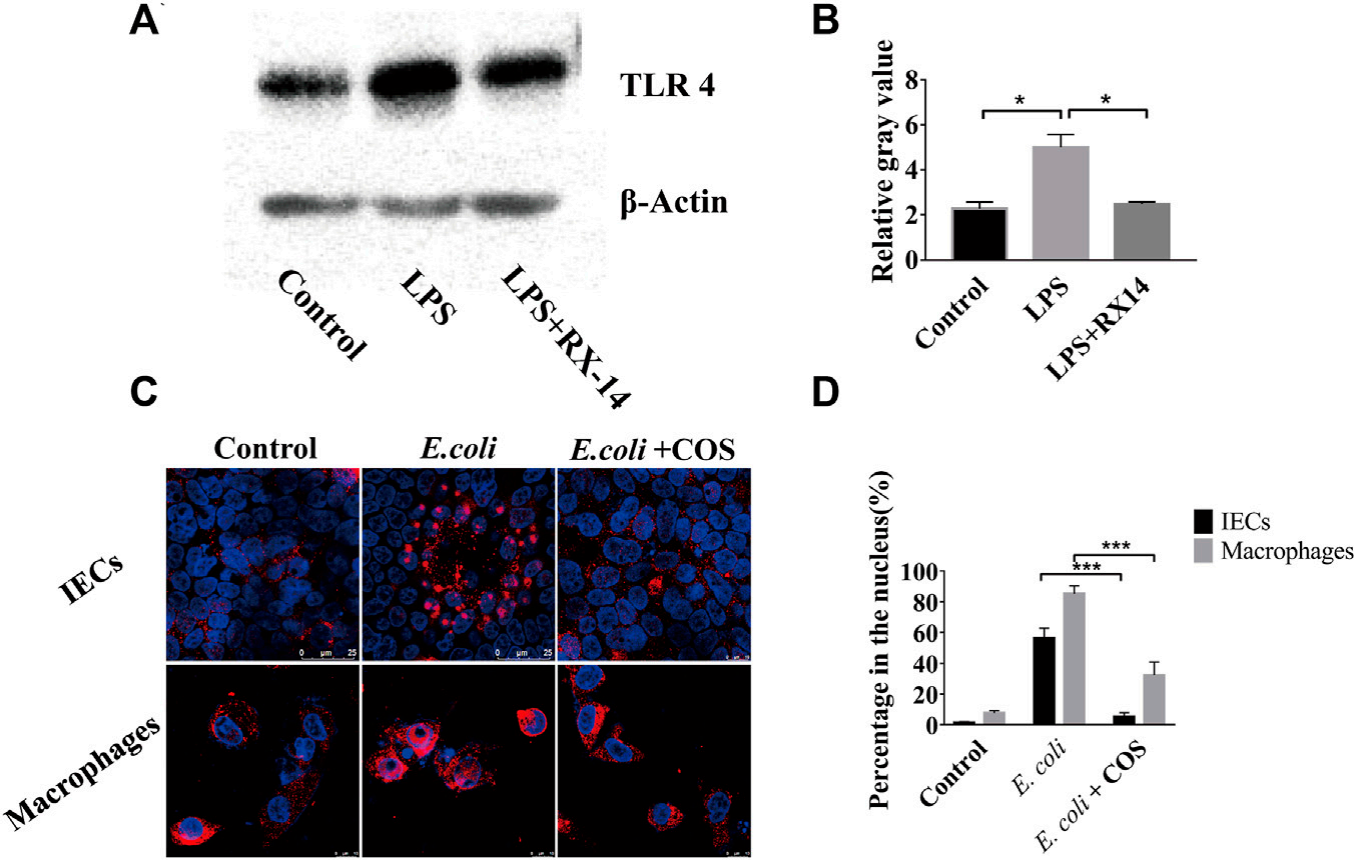
Regulatory effect of COS on NF-κB (p65) signaling pathway in enteritis on a chip. **(A)** The expression of TLR4 detected by Western blot. **(B)** The relative gray value of TLR4. **(C)** Immunofluorescence staining against NF-κB (p65) (Red). **(D)** The binding rate of NF-κB (p65) protein to nuclear DNA. Data were represented as mean ± SD (*n* = 3); One-way ANOVA test was performed, *p < 0.05, ***p < 0.001.

During the development of intestinal inflammation, the activation of the NF-κB signaling pathway has been considered to be the main signaling pathway that causes an excessive inflammatory response (Faber and Baumler, 2014). The TRL 4 protein is the main recognition receptor of *E. coli* that activates the NF-κB signaling pathway. Through WB analysis, it was found that COS can significantly reduce the expression of the TLR 4 protein in intestinal epithelial cells (Figure 6A). After coculturing *E. coli* 11775 with intestinal epithelial cells in the human gut-on-a-chip for 24 h, the expression of the TLR 4 protein increased significantly, and the gray value reached 5.3. When COS were present, the gray value decreased to 2.1 (Figure 6B). By immunofluorescence labeling the NF-κB (p65) protein in intestinal epithelial cells and macrophages, it was found that COS can significantly reduce the binding rate of NF-κB (p65) protein to nuclear DNA (Figure 6C). After 24 h of coculture, the binding rate of NF-κB (p65) protein to nuclear DNA in intestinal epithelial cells and macrophages reached 56.3% and 85.3%, respectively. With COS treatment, binding rates of the nuclear DNAs were 5.3% and 25.7%, respectively (Figure 6D). Through WB analysis, it was found that COS showed little effect on the expression level of NF-κB (p65) (Supplementary Figure S6).

## Discussion

Enteritis is a complex physiological process that includes disorder of intestinal microbiomes, damage of intestinal mucosal and intercellular junction protein, invasion of pathogenic bacteria, accumulation of immune cells, and release of inflammatory factors (Ramos and Papadakis, 2019). Past studies have shown that COS had regulatory effects on gut health and disturbance of gut microbes during the development of mouse enteritis or diabetes (Yousef et al., 2012; Zheng et al., 2018b). However, there are still few reports on the regulatory effects of COS on the damage of intestinal barrier and the inflammatory response during the occurrence and development of human enteritis. Here, for the first time, human gut inflammation on a chip was used to clarify the regulatory effect of COS on the mucus layer, opportunistic pathogens, intestinal epithelial barrier and inflammatory responses during the occurrence and development of human enteritis.

Past studies have proven that the human organ-on-a-chip can simulate the intestinal microenvironment and reproduce intestinal function similar to *in vivo* tissues (Huh et al., 2010; Trietsch et al., 2017). It has become an effective tool for studying the occurrence and development of enteritis, investigating the interaction between intestinal microbes and the host, and exploring the mechanism of drugs repairing enteritis (Kim et al., 2016; Shah et al., 2016; Jalili-Firoozinezhad et al., 2019). The morphological studies proved that human gut-on-a-chip model can form intestinal mucus, dense intestinal epithelial barriers, vascular endothelial barriers, and an immune microenvironment that are similar to physiological states, which can be used to construct models of enteritis disease (Kaser et al., 2010). Our past research has demonstrated that the human gut-on-a-chip can be used to study the interaction between gut microbes and the host, and to explore the effect and mechanism of drugs regulation on enteritis (Jing et al., 2020).

The mucus layer is mainly formed by the connection of complex oligosaccharide chains with backbone proteins through *O*-glycosidic linkages (Hooper and Macpherson, 2010). These glycoproteins are connected through disulfide bonds to form high polymers, which provide a microenvironment for microorganisms to inhabit and as a barrier to protect intestinal epithelial cells (Martens et al., 2018). By introducing DSS into the intestinal lumen, an intestinal injury model *in vitro* was constructed*.* Our results showed that COS can promote expression of mucus layer and had a significant repair effect on damage of mucus barrier induced by enteritis, which haven’t been observed before. Though COS cannot physically adhere to the surface of intestinal epithelial cells, they may provide raw materials for the synthesis of oligosaccharide chains of mucus layer (Reily et al., 2019). In addition, it is known that goblet cells are the main secreting cells of mucous layer, and past study suggested that COS can promote proliferation of goblet cells (Wang et al., 2021).

The integrity of the intestinal epithelial barrier is an important regulator of the occurrence and development of enteritis (Parikh et al., 2019). Our study demonstrated that COS can significantly reduce damage of the intercellular junction of intestinal epithelial cells caused by DSS or *E. coli*, which is consistent with previous report. By using a pathological model of diabetic animals, Zhang et al. (2021) demonstrated that COS can promote the expression of intestinal epithelial intercellular junction protein through the MAPK signaling pathway, thereby improving function of intestinal epithelial barrier (Zheng et al., 2018b). Another possible reason may be that COS can promote expression of mucus layer and reduce the amount of DSS or *E. coli* in contact with intestinal epithelial cells.

The invasion of intestinal pathogenic bacteria can cause excessive inflammation in the intestinal lumen and vascular lumen, and the release of a large amounts of inflammatory factors can lead to damage of intestinal epithelial cells and endothelial cells (Tremaroli and Backhed, 2012). Here, it was found that COS can reduce the amount of *E. coli* adhering to IECs and invading into vascular lumen, which hasn’t been reported before and couldn’t been found in animal models. This may be related to the reason that COS can promote secretion of mucus layer and improve the lubrication effect of intestinal epithelial surface. The deeper mechanism of these results needs to be further explored. Besides, COS can significantly reduce the release of inflammatory factors. This is possible due to the reduction of invading *E. coli* and the regulatory effect of COS on the NF-κB signaling pathway, which is the main one that regulates the occurrence and development of inflammation (Artis, 2008; Maloy and Powrie, 2011). COS can significantly reduce the expression of toll-like receptor 4 (TRL 4), which is the main receptor protein of *E. coli* lipopolysaccharides in intestinal epithelial cells (Cario, 2010). COS can also significantly reduce the nuclear binding law of NF-κB (p65) protein in intestinal epithelial cells and macrophages. These results are consistent with the ones of previous studies (Shi et al., 2019). Yousef et al. (2012) proved that COS can reduce the secretion of TNFα and IL-6 through the NF-κB signaling pathway. In addition, the authors proved that COS can inhibit LPS adhesion and reduce apoptosis of intestinal epithelium caused by inflammatory factors. Gu et al. (2021) proved that COS can decrease the transcriptional levels of proinflammatory cytokines (i.e., IL-1β, IL-8 and TNF-α) and major pathway effectors (i.e., activator protein-1 (AP-1), NF-кB, p38 mitogen-activated protein kinase, c-Jun N-terminal kinase and extracellular regulated kinase). Our studies proved that COS had the ability to reduce inflammatory response in human IBD microsystem for the first time.

## Conclusion

In summary, a human inflammatory bowel disease model caused by DSS or conditional pathogenic *E. coli* was established on a microfluidic chip. This study proved that COS can inhibit the occurrence and development of enteritis by promoting secretion of the mucous layer and reducing the adhesion and invasion of pathogenic bacteria, which were observed for the first time and couldn’t be achieved in animal models. Besides, similar to the results *in vivo*, COS can reduce the inflammatory response through the NF-κB signaling pathway based on this IBD microsystem. An *in vitro* IBD model caused by *E. coli* and a treatment model of IBD by COS were successfully achieved in a human gut-on-a-chip. Our results would point towards a potential application of COS and a drug screening platform in treatment of human IBD.

## Data Availability

The original contributions presented in the study are included in the article/Supplementary Material, further inquiries can be directed to the corresponding authors.
