# Supplementary material for: Chitosan Oligosaccharides Regulate the Occurrence and Development of Enteritis in a Human Gut-On-a-Chip
Source: Front Cell Dev Biol. 2022 Apr 26;10:877892. doi: 10.3389/fcell.2022.877892 (PMC9086312; doi:10.3389/fcell.2022.877892)
Supplement: Supplementary file 1 [file DataSheet1.pdf]

Chitosan oligosaccharides regulate the occurrence and development of enteritis in a human gut-on-a-chip

Bolin Jing[1], Kun Xia[3], Chen Zhang[2], Siming Jiao[2], Limeng Zhu[2], Jinhua Wei[2], Zhuo A Wang[2], Pengfei Tu[1*], Jianjun Li[2*] and Yuguang Du[2*]

[1]State Key Laboratory of Natural and Biomimetic Drugs, School of Pharmaceutical Sciences, Peking University, Beijing 100191, P.R. China.

[2]State Key Laboratory of Biochemical Engineering, Institute of Process Engineering, Chinese Academy of Sciences, Beijing 100190, P.R. China.

[3]The Second Affiliated Hospital, Hengyang Medical School, University of South China, Hengyang 421000, P.R. China.

*** Correspondence:**

Pengfei Tu, Jianjun Li and Yuguang Du

pengfeitu@vip.163.com; jjli@ipe.ac.cn (J.L.); ygdu@ipe.ac.cn (Y.D.)

Supplementary Material

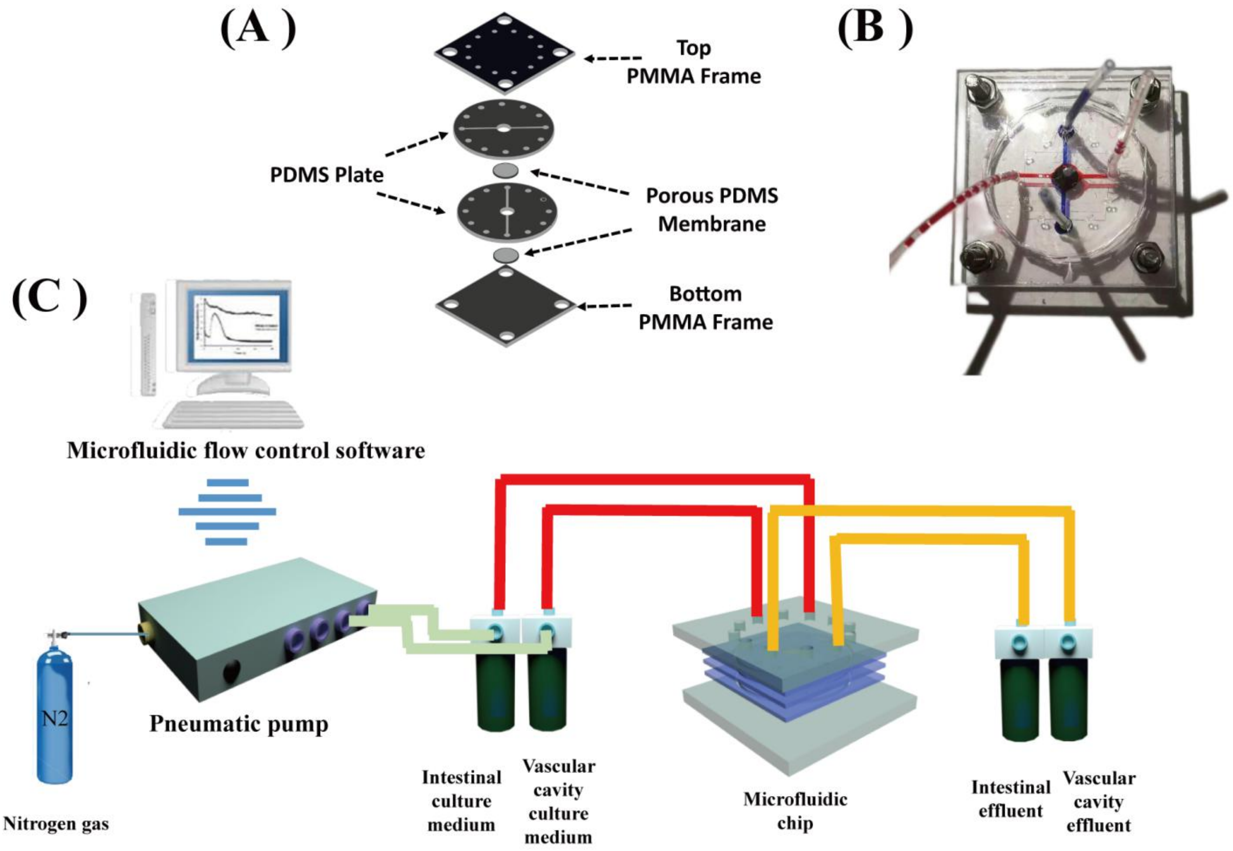

Supplementary Figure 1. Fabrication and assembly of the microfluidic chip. (A)Schematic diagram of the chip structure before assembly. (B) A physical picture of the chip after assembly, blue ink and red ink were poured into the upper and lower channels of the chip, respectively. (C) Diagram of the experimental set-up of the peristaltic human enteritis model with pneumatic pump system for the perfusion of culture media.

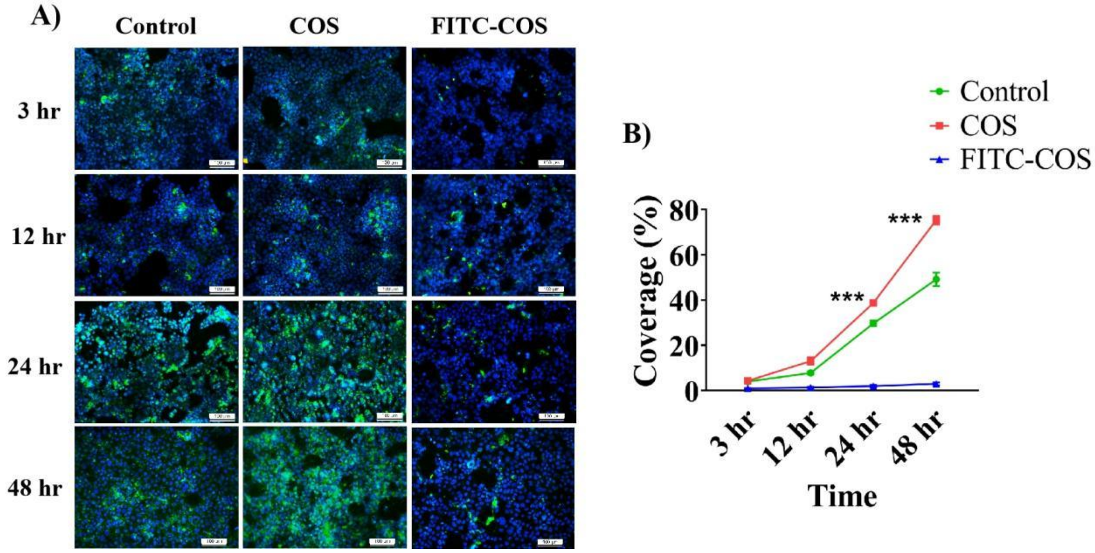

Supplementary Figure 2. COS promote mucus secretion in gut-on-a-chip. (A) Intestinal mucin staining using WGA-FITC (Green), and COS labeled with FITC (Green) adhering to IECs (Right). (B) The coverages of mucin or COS-FITC on IECs over time. Data were represented as mean ± SD (n = 3); One-way ANOVA tests were performed, ***$P < 0.001$ vs control group.

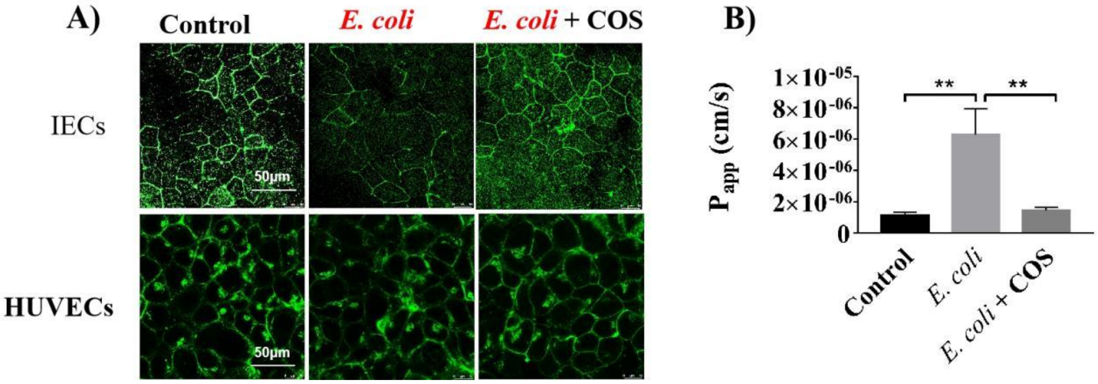

Supplementary Figure 3. Repair effects of COS on barrier injury of intestines and blood vessels induced by *E. coli*. (A) Immunofluorescence staining against occludin on IECs and CD144 on HUVECs. (B) The apparent permeability of FITC-dextran (4 kDa) across intestinal epithelium. Data were represented as mean ± SD (n = 3); One-way ANOVA test was performed, *P < 0.05, ***P < 0.001, ****P < 0.0001.

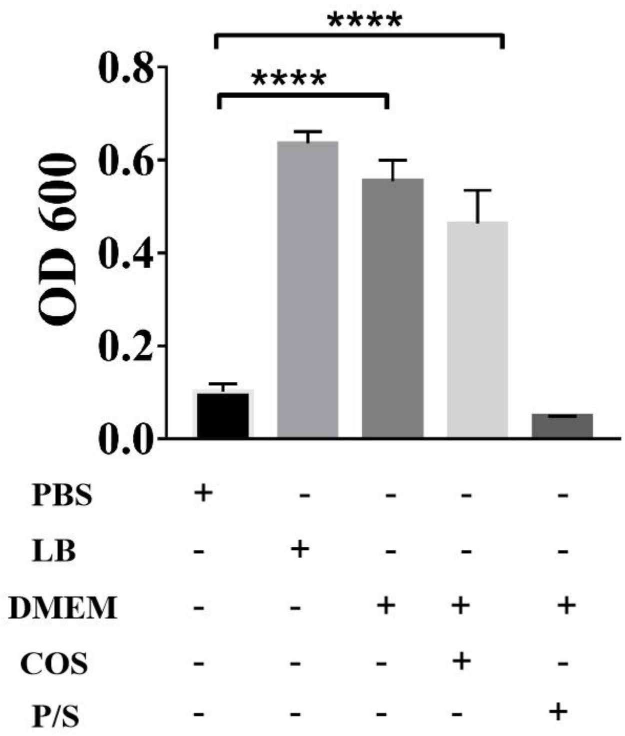

Supplementary Figure 4. Growth of *E. coli* under different culture conditions, PBS represent phosphate buffer solution, LB represent lysogeny broth culture medium， DMEM represent dulbecco' modified Eagle's medium, COS represent chitosan oligosaccharides, P/S represent penicillin and streptomycin. Data were represented as mean ± SD (n = 3); One-way ANOVA test was performed, ****P < 0.0001.

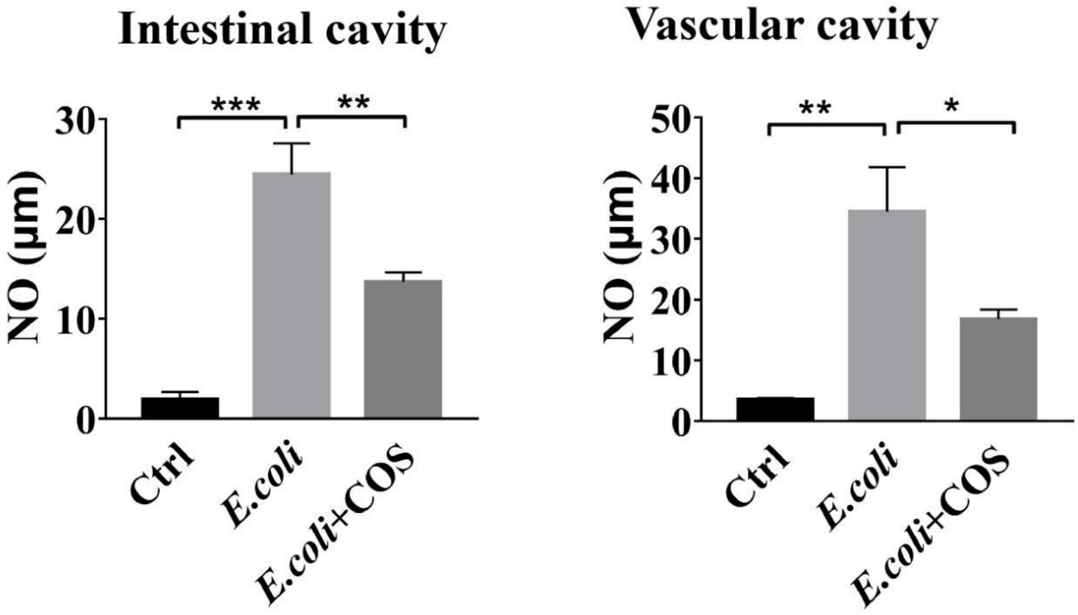

Supplementary Figure 5. The concentration of NO in the outflow solution from enteritis on a chip. Data were represented as mean ± SD (n = 3); One-way ANOVA test was performed, *P < 0.05, **P < 0.01, ***P < 0.001.

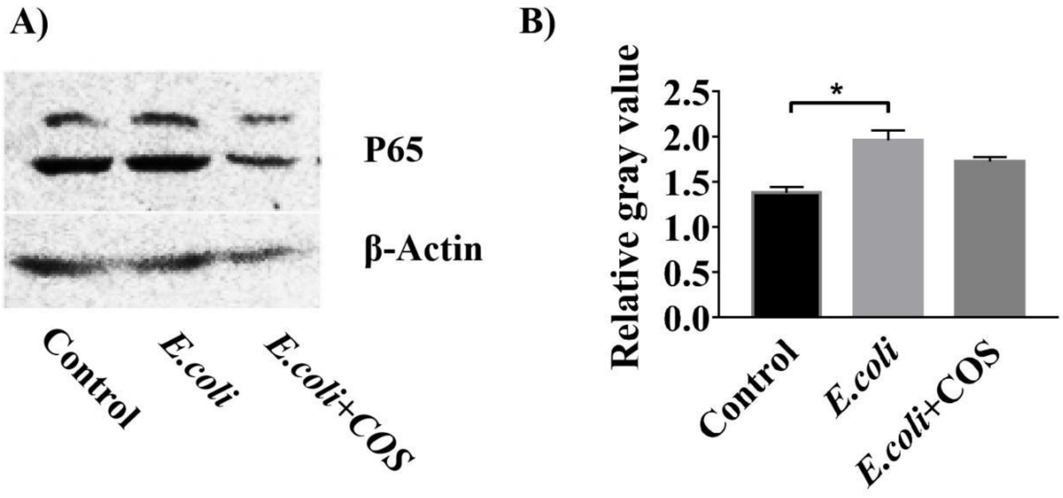

Supplementary Figure 6. (A) The expression of NF-κB (p65) detected by Western blot. (B) The relative gray value of NF-κB (p65). Data were represented as mean ± SD (n = 3); One-way ANOVA test was performed, *P < 0.05.
